# Metacognition vulnerabilities in time of crisis: Who to protect from suicidal risk?

**DOI:** 10.1002/brb3.2794

**Published:** 2022-11-11

**Authors:** Sylvia Martin, Anna Oltra, Jonathan del Monte

**Affiliations:** ^1^ Psycho.TCCE Private Practice Nîmes France; ^2^ Center for Research and Bioetchics Uppsala University Uppsala Sweden; ^3^ Private Practice Toulouse France; ^4^ Psychosocial Laboratory Aix‐Marseille and Nîmes Universities Nîmes France

**Keywords:** COVID‐19, general population, hopelessness, Metacognition, suicide risk

## Abstract

**Introduction:**

During stressful events, we are all trying to cope. We may not be equal depending on our emotional, psychological, and mental states. During the COVID‐19 pandemic, we could try to avoid negative information processing and anxiogenics content to prevent unhealthy thinking processes. One of the processes we can observe regarding our way of thinking and its impact on our psychological well‐being is Metacognition.

**Methods:**

We recruited 104 outpatients in 2018. In 2020, during the pandemic, we recruited 216 outpatients and 176 healthy controls. We assessed their level of metacognition with the MCQ30 scale together with Suicidal risk and Hopelessness.

**Results:**

All three groups showed significant differences, with the nonclinical sample having higher scores in MCQ30. Regression revealed the different profiles where Hopelessness was the only predictor for the clinical sample, whereas metacognition was an adjunctive predictor of suicidal risk for the nonclinical sample.

**Conclusion:**

Our results showed that the COVID‐19 crisis influenced metacognitive levels for the nonclinical sample but not for the clinical population. Moreover, Hopelessness predicted suicide risk for both populations, but Metacognition was also a predictive factor for the nonclinical sample. We conclude with the possible impact of preventive measures based on Metacognitive work that can be created out of these results.

## Graphical abstract

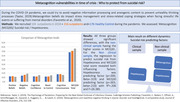

## 1 INTRODUCTION

During stressful events, we are all trying to cope, and the last years put us all at risk to suffer psychological stress. We may not all be equal depending on our emotional, psychological, and mental states. As Lee (2020) commented, we can stay safe during the crisis by avoiding too much information processing and being exposed to media's anxiogenics content. Nevertheless, the objective of such a recommendation is mainly to help people avoid creating or encouraging unhealthy thinking processes. As Taylor proved in 2019, a significant level of information can prevent harm, but too much can be disturbing and increase dysfunctional thinking.

One of the thinking processes we can monitor in order to check if we experience dysfunctional thinking is Metacognition. Wells based his definition of Metacognition on the previous conceptualization of it as being “*that part of cognition that is responsible for appraising, monitoring and controlling thinking (* *Flavell*, *1979* *). It consists of knowledge and beliefs about thinking, implementation of strategies of mental regulation and appraisals and judgments of mental status. It biases and controls the selection and execution of appraisals, attention and memory*” (p. 134) (Flavell, 1979 cited in Wells, 2010). Amidst different metacognition models, Wells's had been the most important one to relate metacognition to psychiatric disorder from general anxiety disorders (Wells, 2006) to post‐traumatic disorders (Roussis & Wells, 2006) and obsessional‐compulsive disorder (Myers et al., 2009) in anxiety but also to depression (Papageorgiou & Wells, 2003). The model explains that when a situation or a stimulation triggers the worry, the first type of worry will be activated based on “positive meta‐beliefs” that then will activate “negative meta‐beliefs” in cascade, which will then create a meta‐worry “Type 2 worry” that will influence emotional, behavioral, and cognitive response (see Wells, 1999). We understand why metacognition can be essential for behavioral adaptation. Patients need to have complex consciousness about their ideas and feelings to implement change, engage in recovery processes, and find motivation for implementing change (Landine & Stewart, 1998; Zimmerman & Moylan, 2009).

A large body of research in psychology demonstrated that Metacognition plays an important role in cognitive tasks, everyday behavior, and problem‐solving. Amidst clinical psychology, Metacognition is known as an executive and a neuropsychological function linked to clinical outcomes in hard‐to‐treat disorders, such as psychosis, bipolar disorder, and resistant anxiety disorders (David et al., 2012; Jacobi et al., 2006; Moritz et al., 2010a; Moritz et al., 2010b; Myers & Wells, 2005; Tas et al., 2014; Van Oosterhout et al., 2016; Varese & Bentall, 2011). A new direction for research and treatment started to implement metacognitive work as a critical factor in mood disorders and personality disorder (Normann et al., 2014; Teasdale et al., 2002; Wells, 2011).

### 1.1 Suicidal vulnerable populations and COVID‐19

Some populations are more at risk of suicidal risk. Among, psychiatric patients are seen as high risks (Balázs et al., 2013; Handley et al., 2018; Krysinska & Lester, 2010; Park et al., 2018; Stanley et al., 2018).

Extensive literature came out during this COVID‐19 pandemic, advertising for the possible increase in suicidal risk (Gunnell et al., 2020; McIntyre & Lee, 2020; Nicola, 2020; Niederkrotenthaler et al., 2020; Sher, 2020) or measurements of increased suicidal risk (Caballero‐Domínguez et al., 2022; Czeisler et al., 2020; Halford et al., 2020; Iob et al., 2020; Kahn et al., 2020; Thakur & Jain, 2020).

Metacognition beliefs do impact stress management and stress‐related coping strategies when facing stressful life events or suffering from mental disorders (Aydin et al., 2020; Oguz et al., 2019; Wells & Carter, 2009; Wells et al., 2009; Yazihan et al., 2019) and can be linked to suicidal risk (Abdullah, 2018; Aghajani & Samadifard, 2020; Cesur et al., 2019; Hallard, 2017).

Our study aims to investigate the differences in metacognitive processes and their relation to suicide risk among at‐risk populations and healthy control to see if the potential mechanism of suicide was the same when people––with or without mental illness––were facing the COVID‐19 pandemic.

## 2 METHODS

### 2.1 Participants

We recruited 104 patients in 2018 before their inclusion in treatment in an outpatient daycare therapy unit. Patients were addressed for anxiety and depressive disorder (diagnosed from DSM 5 criteria excluding comorbidities of psychotic disorders or personality disorders) by their psychiatrist (mean age for comparative sample = 21.15, SD = 20.708). We recruited 176 healthy controls for the control group during the COVID‐19 crisis (mean age = 34.7, SD 12.181). We also collected data from 216 outpatients during the crisis (mean age = 32.39, SD = 13.931). Exclusion criteria for both groups were: (1) known neurological disease and (2) developmental disability. All participants were proficient in the French language, had a normal or corrected‐to‐normal vision, and were naïve about the purpose of the study. The present protocol follows Helsinki's Ethics recommendations. Participants gave written consent to participate in this experiment. The capability to provide informed consent was established through a structured interview and confirmed by their treating psychiatrists. Both psychiatrists and psychologists in charge of the study confirmed the differential diagnoses of anxiety disorder and depression disorder, excluding other comorbidities. We measured clinical scores but also demographic elements: the sense of isolation (“Do you feel more isolated now?” on a Likert scale with 1 = Not at all/5 = Yes, very often), number of people being in contact with (“Since the confinement, how many people do you have regular contact with (2x / week minimum, whether face to face, via phone or social networks)?”), number of people isolated with (“How many people do you live with during the lockdown?”), satisfaction about relational life (“What has been the overall impact of the lockdown on your relationship life?” on a Likert scale with 1 = Negative and 5 = Positive), and satisfaction about emotional life (“What was the overall impact of the lockdown on your emotional life?” 1 = Negative and 5 = Positive).

### 2.2 Measures

#### 2.2.1 Metacognitive questionnaire MCQ30

We used the 30‐item version of the MCQ from Wells and Cartwright‐Hatton (2004). The questionnaire comprises five distinct factors: (1) positive beliefs about worry (or Positive beliefs); (2) negative beliefs about thoughts concerning uncontrollability and danger (or Negative beliefs); (3) cognitive confidence (assessing confidence in attention and memory); (4) negative beliefs concerning the consequences of not controlling thoughts (or Need or control); and (5) cognitive self‐consciousness. Alpha reliabilities for the five subscales range from 0.72 to 0.89. A higher score means that there are more metacognitive issues.

#### 2.2.2 SBQR

SBQR (Shakeri et al., 2015) is one of the only Suicidal risk scales asking about future anticipation of suicidal thoughts or behaviors, past and present ones, and includes a question about lifetime suicidal ideation, plans to commit suicide, and actual attempts. A total score of 7 and higher in the general population and a total score of 8 and higher in patients with psychiatric disorders indicate a significant risk of suicidal behavior. The Cronbach's alpha for the SBQ‐R items was 0.80 (French version: Potard et al., 2014).

#### 2.2.3 H

Beck's Hopelessness Scale (Beck et al., 1989; French translation Bouvard et al., 1992). A 9‐point clinical rating scale was used to assess the Hopelessness's severity. A cutoff score of 6 or above predicted nine (90.0%) of those committing suicide. The Cronbach's alpha is 0.72.

### 2.3 Statistical analysis

Pearson parametric correlations were used to explore the relationships between clinical data as the variables are normally distributed. We applied multiple linear regression to estimate the shared covariance. For all analyses, the level of significance was set to *p* < .05 (*), *p* < .005 (**), and *p* < .001 (***).

## 3 RESULTS

### 3.1 Descriptive analysis

We created three groups, C18 for the clinical sample before COVID‐19, NC19 represents the nonclinical sample during the COVID‐19 crisis, and C19 for the clinical sample at the time. For comparison purposes, we will use literature data to understand the nonclinical sample score before the crisis, which will be defined as NC18 (*n* = 119).

Across the sample, MCQ30 were ranging from 68.21 (SD = 14.28) for NC19 to 76.28 (SD = 14.22) for C18. On suicidal risk, the SBQR score ranged from 5.23 (SD = 2.55) for NC19 to 7.30 (SD = 3.77) for C19, and the Hopelessness scale went from 5.59 (SD = 4.16) for NC19 to 7.10 (SD = 4.66) for C19 (no data for C18). All means are reported in Table 1.

**TABLE 1:** Mean table

Group	NC19		C19		C18	
	Mean	SD	Mean	SD	Mean	SD
Age	32.39	13.93	34.70	12.18	21.15	20.708
SBQR	5.23	2.55	7.30	3.77		
H	5.59	4.16	7.10	4.66		
Positive beliefs	12.51	4.49	12.89	4.52	11.40	4.8
Negative beliefs	14.14	4.90	15.99	4.51	18.72	4.49
Cognitive confidence	12.40	5.09	12.36	5.02	15.33	5.43
Need of control	11.87	3.83	12.17	4.15	14.98	4.17
Cognitive consciousess	17.29	3.34	17.64	3.32	15.85	3.74
MCQ30Tot	68.21	14.28	71.06	12.63	76.28	14.22
Sense of isolation	2.73	1.277	7.92	4.606		
Number of people being in contact with	8.12	7.540	7.48	3.878		
Number of people being isolated with	2.13	1.749	8.66	3.838		
Satisfaction about relational life	2.86	.945	1.69	1.425		
Satisfaction about emotional life	2.72	.972	3.60	3.040		

Abbreviations: C18, clinical sample before COVID‐19; C19, clinical sample during COVID‐19; H, Hopelessness; NC19, nonclinical patients during COVID‐19; SBQR, Suicidal behavior questionnaire.

p < .05 (*), p < .005 (**), and p < .001 (***).

### 3.2 Comparisons

**FIGURE 1:**
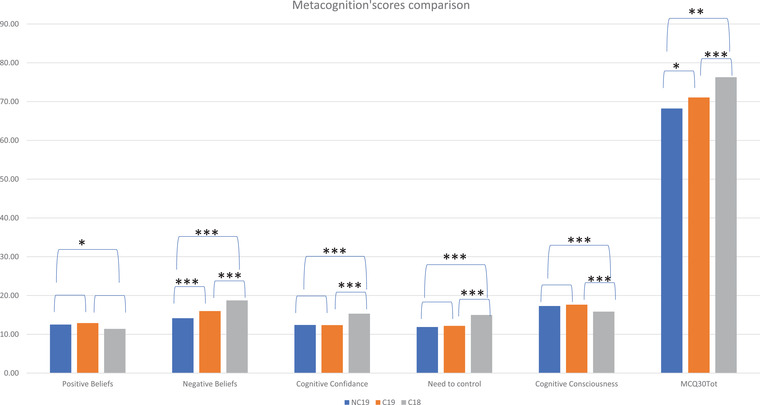
Comparison of Metacognition levels

Differences using *t*‐test U revealed differences between groups regarding clinical dimensions except for C18, where we compared only metacognitive profiles (Figure 1).

Differences were found between C19 and NC19 scores regarding H *t*(354,344) = −3,32*** with Cohen's *d* = 0.34, Negative beliefs *t*(378,605) = −3,839*** with *d* = 0.39, MCQ30 total *t*(380,410) = −2,072* with Cohen's *d* = 0.21, and for SBQR *t*(299,251) = −6,197*** with Cohen's *d* = 0.64.

*T*‐test comparing C19 and C18 showed differences regarding all MCQ30 dimensions and global score but was not revealing differences for Positive beliefs dimensions (Table 2). But all MCQ30 measurements of NC19 compared to C18 were significantly different.

**TABLE 2:** *T*‐test comparison table with C18 for MCQ30

	NC19				C19			
	t	df	Bilateral significance	Cohen's d	t	df	Bilateral significance	Cohen's d
Positive beliefs	2.56	206.10	.011*	0.15	1.95	194.62	.052	NS
Negative beliefs	–4.90	217.03	.000***	0.61	–8.22	223.24	.000***	0.97
Cognitive confidence	–4.53	202.74	.000***	0.50	–4.57	195.02	.000***	0.89
Need of control	–5.45	215.35	.000***	0.67	–6.37	191.57	.000***	0.77
Cognitive consciousness	4.04	196.20	.000***	0.50	3.32	186.93	.001***	0.40
MCQ30Tot	–3.09	196.27	.002**	0.38	–4.71	207.27	.000***	0.56

Abbreviations: C19, clinical sample during COVID‐19; NC19, nonclinical patients during COVID‐19.

p < .05 (*), p < .005 (**), and p < .001 (***).

Metacognition scores for the nonclinical sample appear to be increased for NC19 compared to the weighted scores from literature samples that we can consider as NC18 (*N* = 119) for all subdimension's means (Wells & Cartwright‐Hatton, 2004). Looking at the Positive beliefs mean, it varied from 9.6 Expected Mean (EM) (SD = 3.46, Cohen's *d* = 0.722), Negative beliefs from 9.3 EM (SD = 2.79; Cohen's *d* = 1.13), Cognitive confidence mean from 9.51 EM (SD = 4.06; Cohen's *d* = 0.627), Need of control mean from 8.34 EM (SD = 2.6, Cohen's *d* = 1.078), and Cognitive confidence mean from 11.65 EM (SD = 4.68, Cohen's *d* = 1.389).

### 3.3 Correlation analysis

For C19, the SBQ‐r score was positively correlated to H (*r* = 0.547**), Negative beliefs (*r* = 0.253**), Need for control (*r* = 0.301**), and Total MCQ30 score (*r* = 0.202**). H also correlated to Negative beliefs (*r* = 0.365**), Need for control (*r* = 0.365**), and Total MCQ30 score (*r* = 0.242**).

For NC19, SBQ‐r correlated positively with H (*r* = 0.413**), Positive beliefs (*r* = 0.149*), Negative beliefs (*r* = 0.276**), Cognitive confidence (*r* = 0.281**), Need of control (*r* = 0.285**), Cognitive consciousness (*r* = 0.147*), and Total MCQ30 score (*r* = 0.353**). H also correlated to Positive beliefs (*r* = 0.228**), Negative beliefs (*r* = 0.394**), Cognitive confidence (*r* = 0.334**), Need of control (*r* = 0.418**), and Total MCQ30 score (*r* = 0.449**).

### 3.4 Regression analysis

For NC19, the regression to predict suicidal risk from H and MCQ30 total score revealed both influencing factors. H was predicting factor (*p* = .000, beta = 0.319), and MCQ30 predicting factor (*p* = .003, beta = 0.210) with *R* ^2^ = 0.206 and *F* = 26.385.

For C19, running the same regression, only H was a predicting factor (*p* = .000, beta = 0.529) with *R* ^2^ = 0.305 and *F* = 37.949.

## 4 DISCUSSION

The comparison between C19 and NC19 revealed differences in Suicidal risk, H, Negative beliefs, and MCQ30 total score (C19 having the highest score), which is quite relevant as C19 are supposed to have difficulties with mood and behaviors (Martín et al., 2014; Konstantellou & Reynolds, 2010; Stainsby & Lovell, 2014).

For C19, there were differences from C18; all the metacognitive scores were different on a significant level except for Positive beliefs and Cognitive consciousness (C18 being the highest in all dimensions and total scores). It can be interpreted as the metacognitive abilities that were impaired for C19 mainly being under control due to their care except for positive beliefs and cognitive consciousness dimension that were increasing. Another explanation could be, referring to Wells's model, that Positive beliefs about worry are the first level organizing subsequent negative beliefs and meta‐worry system, therefore, we could hypothesize that the first level of Type 1 worry may be the same (no difference on *t*‐test), and the other levels of metacognition may be different on a crisis context as the Type 1 worry is related to emotion already on the first steps, so emotional level created by the stressful context did change the metacognitive functioning (Wells, 1999).

For NC19 comparison with C18, C18 were higher on score except for Positive beliefs and cognitive consciousness like for C19.

Indeed, our comparison with literature results showed increased Metacognition scores for our NC19 compared to NC18, suggesting that the nonclinical sample's Metacognition could be more sensitive to crisis times. This is congruent with the original research on MCQ30, where the Metacognition increase was related to indices of emotional disorder (Wells & Davies, 1994) and acute stress disorder (Warda & Bryant, 1998). During the pandemic in France, some data revealed the negative impact of the lockdown situation on the mental health of the general French population (Peretti‐Watel et al., 2020), the increased consumption of antidepressants and antipsychotics (+21.6% and +21.5%, respectively) (Weill et al., 2020), and the decrease in the psychiatric emergency venues compared to previous years (patient's postponing their consultation because of the fear of the virus and increased anxiety, not due to well‐being) (Pham‐Scottez et al., 2020).

Correlation Intra‐group, for NC19 or C19 group, showed almost the same array of correlations but depending on the group, the predictive impact of Hopelessness and Metacognition on Suicide risk was different. For NC19, Hopelessness and Metacognition were predictors, whereas, for C19, only Hopelessness was a predicting factor. These results suggest that, when facing a global crisis, the nonclinical population could be more at risk of Suicide. Our results suggest that they were facing more psychological issues than the clinical sample regarding Metacognition which influence their Suicidal behavior risk.

It looks like no surprise that some metacognitive dimensions could relate to anxiety levels. In their research, Melli et al. (2018) found that specifically, Negative beliefs predicted health anxiety symptoms over‐and‐above depression, general anxiety, anxiety sensitivity, and health‐related dysfunctional beliefs. These results encourage the hypothesis that Metacognition may have an essential role in health anxiety in clinical samples. In psychiatric disorders, Negative beliefs, Cognitive confidence, and Need to control thought were heightened (Favaretto et al., 2020). Metacognition could be of interest for nonclinical samples also as MCQ30 scales is considered as an assessment of vulnerability to and maintenance of emotional disorders in nonclinical samples (Martín et al., 2014; Baptista et al., 2020; Hassanvand Amouzadeh & Roshan Chesly, 2013). These elements confirm that Metacognition is highly related to executive functioning that may be differently impaired in psychiatric disorder but also variate among general population individuals as an important factor for self‐regulation (Roebers, 2017; Lyons & Zelazo, 2011; Sun et al., 2017).

For example, metacognitive issues can have a deleterious effect on behavior when feeling anxious in both clinical and nonclinical populations. For example, Metacognition has a role in anger, aggression (Salguero et al., 2020), and paranoia in clinical and nonclinical samples (Kaltsi et al., 2018; Sellers et al., 2017).

In the past few years, many so‐called “Metacognitive Therapies” have been created to treat a vast range of psychiatric disorders, from schizophrenia to depression and anxiety disorders (Lysaker et al., 2020a; Hoffart et al., 2018; Moritz et al., 2019; Brown et al., 2021). Further research needs to assess its potential impact on preventive measures regarding nonclinical samples. In the nonclinical sample, metacognition has been recently related to psychological health, so preventing actions can be up to consider (Aydin & Kaynak, 2021).

Literature starts to clearly define which type of Metacognitive Therapy could be useful for certain therapeutical goals, from having the patient be aware of their cognitive biases (Metacognitive Training) or focusing more on attention's functions (Metacognitive Therapy) to get the patient to be mindful about their own and other thoughts (Metacognitive‐oriented integrative therapies) (for a review, see Philipp et al., 2020).

There is a wide range of possible effects across Cognitive Behavioral Therapy (CBT) and Meta‐cognitive approaches available for the clinical population. Moritz et al. (2018) highlighted that even if they share common principles, CBT, Metacognitive Therapy, Metacognitive Training, and MERIT (the most renown Metacognition Therapy) differ. Some are more centered on exposure, emotional biases’ elicitation, dysfunctional coping strategies, or specific disorder‐related cognitive distortion.

Recent encouraging results proved the effectiveness of Metacognitive work on stress and anxiety levels in people facing health concerns. For example, Fisher et al. (2019) showed effects on cancer patients who received six Metacognitive Centered Therapy sessions with a promising 80% of recovered participants post‐treatment. These elements stand as a possible brief transdiagnostic psychological intervention to address psychological stress in a heterogeneous group of participants. Moreover, the first meta‐analysis on Metacognitive Therapy proves that it has a significant impact on anxiety and depression, but trials need to go for larger sample sizes (Normann & Morina, 2018).

In nonclinical and clinical populations, controlling for current depression and anxiety symptoms, a decreased ability to shift between mental states was associated with increased Negative beliefs and a Need to control dimensions of the MCQ30 scale. The results suggest a fundamental association between Metacognition and executive control. Individual differences in executive control could improve critically in metacognitive therapy's personalization effects (Kraft et al., 2017).

The overall effect of Metacognition Ther and CBT on resistant anxiety is also encouraging, as Hoffart et al. (2018) showed that coping activities, Negative beliefs, and Positive beliefs decreased throughout treatment. Negative and Positive beliefs levels dropped more in Metacognition Therapy than in CBT.

Recent research development proves that metacognitive intervention for psychiatric illness can be effective (Lysaker et al., 2020b), but further research needs to assess its possible protective impact on suicidal risk in a time of crisis for nonclinical populations. In their mini‐review from Raj et al. (2021), the authors suggested serious work on these cognitive factors to reduce suicide: mindfulness, interpersonal psychotherapy, and cognitive behavior therapy. Further research could work on their implementation to prevent harmful stress for clinical and nonclinical populations. A recent study tried to implement the Metacognition level as a good tool for routine clinical measurement. It can give good information about social and psychological functioning and help determine subjective recovery experience (Dawood et al., 2021).

### 4.1. Limits

Our research contains several limitations. The main limitation is that due to the data collected from other research settings, we could not measure Hopelessness and SBQ‐r score from the clinical sample from C18. Another limitation is the sample selection that did not properly separate anxious and depressed patients as the referring psychiatrist was confirming anxio‐depressive issues instead of referring to accurate diagnosis but was only confirming that patient had no history of other axis 1 disorder nor personality disorders. The last limitation was due to online recruitment that may have been biased due to the extreme stress experienced at the time by groups NC19 and C19 (Schaurer & Weiß, 2020). Finally, as the research was not focusing on the direct effects of COVID on mental health, so our demographic data could just indirectly address emotional issues at the moment but not verify if the effect was specifically due to COVID (e.g., being ill themselves, mourning a disease relative due to COVID, having a family member in the intesive care, or having long‐lasting effects of the COVID) even if COVID has been related to different levels of depression or demoralization (Shader, 2020).

## 5 CONCLUSION

Slight differences exist in Metacognitive scores, even if clinical measures differ significantly between the groups. The main results of our research lay in the fact that Metacognitive issues can predict suicidal risk among nonclinical populations in time of global crisis like the COVID‐19 pandemic. The specific Metacognition factor could be an essential factor to assess directly. Metacognitive intervention could benefit global distress prevention as some research has already clarified the possible impact of a metacognitive intervention on mental and physical health issues (see Capobianco et al., 2020 for a review). These results can encourage preventive measures to prevent suicide risk in non clinical sample by developing metacognitive skill training or implementing metacognitive therapy.

## AUTHOR CONTRIBUTIONS

SM made substantial contributions to the conception and design of the work; the acquisition, analysis, interpretation of data; drafted the work, revised it critically for important intellectual content; approved the version to be published; and agree to be accountable for all aspects of the work in ensuring that questions related to the accuracy or integrity of any part of the work are appropriately investigated and resolved.

AO made substantial contributions to the conception, revised it critically for important intellectual content; approved the version to be published; and agree to be accountable for all aspects of the work in ensuring that questions related to the accuracy or integrity of any part of the work are appropriately investigated and resolved.

JDM made substantial contributions to the conception of the work and revised it critically for important intellectual content; approved the version to be published; and agree to be accountable for all aspects of the work in ensuring that questions related to the accuracy or integrity of any part of the work are appropriately investigated and resolved.

### PEER REVIEW

The peer review history for this article is available at: https://publons.com/publon/10.1002/brb3.2794

## Data Availability

All data will be available under reasonable demand to the corresponding author.
